# A scoping review of the application of telemedicine in home-based palliative care patients: insights for healthcare systems with emphasis on the Chinese context

**DOI:** 10.3389/fpubh.2026.1788751

**Published:** 2026-02-20

**Authors:** Chen Zhou, Meiwan Zhang, Hannan Dong, Lichun Xing, Dongying Li

**Affiliations:** 1 Department of Intensive Care Unit, The Second Affiliated Hospital of Nanchang University, Nanchang, China; 2 School of Nursing, Jiangxi Medical College, Nanchang University, Nanchang, China

**Keywords:** home-based care, hospice care, palliative care, scoping review, telecare, telemedicine

## Abstract

**Objective:**

To conduct a scoping review on the fundamental aspects, application effectiveness, and existing problems of telemedicine in home-based palliative care (HBPC) patients, with the aim of providing evidence-based insights for future practice and research, particularly for healthcare systems such as China’s that are in the developmental stages of HBPC.

**Methods:**

Relevant studies were systematically retrieved from databases including PubMed, Web of Science, Cochrane Library, Embase, ScienceDirect, CNKI, Wanfang Data, VIP, and CBM from their inception to February 2, 2026.using a comprehensive set of keywords related to telemedicine, home-based care, and palliative care. Included studies were summarized and analyzed.

**Results:**

A total of 18 studies were included. Telemedicine applications in HBPC are diverse, encompassing forms such as applications and video conferencing. Their core content covers six key elements, including symptom management and psychological support. Telemedicine demonstrated positive effects in improving patients’ symptom burden, quality of life, and optimizing healthcare resource utilization.

**Conclusion:**

Telemedicine, as a feasible, effective, and cost-efficient supplementary model for HBPC, shows great potential. For contexts like China, Future efforts should focus on conducting high-quality research, improving policy support and talent cultivation, and enhancing cultural adaptability to promote its standardized development and large-scale application.

## 1 Introduction

In recent years, the aging population in China has continued to deepen, leading to an increasingly urgent societal demand for high-quality palliative care (1). However, China ranks only 53rd globally in the quality of death for terminal patients (2), highlighting the urgency for development in this field. Palliative care, centered on patients and caregivers, emphasizes symptom control and dignity maintenance, effectively enhancing the quality of life at its end stage (3). Home-Based Palliative Care (HBPC) is a service model within palliative care that aims to provide comprehensive care for terminal patients in a home environment, alleviating their physical, psychological, social, and spiritual suffering, and helping them pass away peacefully and with dignity (4). It is recognized as a high-quality end-of-life care model (5). Research (6–8) indicates that HBPC can improve patients’ social support systems, alleviate psychological distress, reduce medical costs, and increase satisfaction among patients and their families. Currently, HBPC in China is still in its early stages of development, primarily relying on home visits and telephone follow-ups by healthcare professionals. This approach is limited by time and geography, often resulting in generally low patient compliance (9). With the advancement of information technology, telemedicine has gradually become an important direction. It can overcome temporal and spatial limitations, provide personalized diagnosis and treatment (10–13), and offers advantages such as optimizing resource allocation and enhancing care continuity (14), thus providing a new pathway for HBPC development. This review is conducted with a specific interest in deriving insights applicable to healthcare systems, like China’s, that are seeking to develop or enhance their HBPC services. While Foreign countries have conducted numerous studies in the field of remote HBPC, research and practice within China are still in their infancy. The application forms, content, and effectiveness evaluation of telemedicine in HBPC remain unclear, with significant heterogeneity among studies. Therefore, this study employs a scoping review methodology to systematically review relevant literature, aiming to provide an evidence-based reference for the standardized application and development of telemedicine in home-based palliative care, with considerations for adaptation in healthcare systems like China’s.

## 2 Information and methods

### 2.1 Defining the research questions

The research questions are: (1) What are the application forms of telemedicine in HBPC patients? (2) What content elements are included in telemedicine-based interventions? (3) What is the application effectiveness of telemedicine for HBPC patients, and what are the corresponding evaluation indicators?

### 2.2 Search strategy

The search strategy was developed in consultation with a medical librarian. To ensure a comprehensive literature review, search strings were constructed using a combination of Medical Subject Headings (MeSH) and free-text keywords corresponding to three core concepts: (1) telemedicine/telehealth/telecare/remote care, (2) home-based care, and (3) palliative care/hospice care. We referenced keywords from existing reviews on similar topics to enhance sensitivity. Searches were conducted from the inception of each database until November 22, 2025, across the following electronic databases: PubMed, Web of Science, Embase, Cochrane Library, CINAHL, CNKI, Wanfang Database, and the Chinese Biomedical Literature Database (CBM). Manual searching of reference lists from key articles was also performed to identify additional relevant studies. No restrictions were applied regarding language or publication status. Taking PubMed as an example, the search strategy was as follows: [“Telemedicine” (MeSH Terms) OR “Telehealth” (MeSH Terms) OR “Remote Consultation” (MeSH Terms)] OR (“mobile health” (Title/Abstract) OR “mHealth” (Title/Abstract) OR “eHealth” (Title/Abstract) OR “digital health” (Title/Abstract) OR “telecare” (Title/Abstract) OR “remote care” (Title/Abstract) OR “virtual care” (Title/Abstract)] AND {“Home Care Services” (MeSHTerms) OR [“Home” (Title/Abstract) OR “Home-Based” (Title/Abstract) OR “home care” (Title/Abstract)]} AND {“Palliative Care” (MeSH Terms) OR “Hospice Care” (MeSH Terms) OR [“palliative care” (Title/Abstract) OR “hospice care” (Title/Abstract) OR “terminal care” (Title/Abstract) OR “end of life care” (Title/Abstract)]}.

### 2.3 Inclusion and exclusion criteria

*Inclusion criteria*: (1) Study participants were HBPC patients; (2) The intervention involved applying various telemedicine technologies to provide services and support for HBPC patients; (3) Study types included randomized controlled trials (RCTs), quasi-experimental studies, mixed-methods studies, etc.

*Exclusion criteria*: (1) Interventions were not specific or consisted solely of phone calls, text messages, or emails; (2) Non-Chinese or non-English literature; (3) Full text unavailable; (4) Literature types such as study protocols, guidelines, reviews, etc.

### 2.4 Literature screening and data extraction

**Figure 1:**
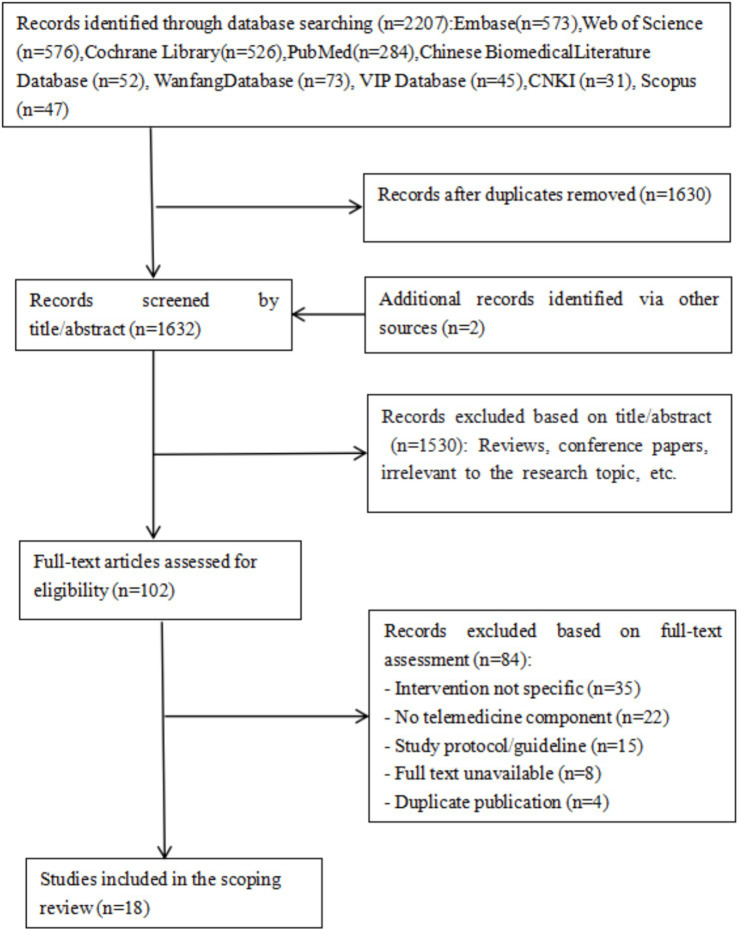
Flow chart of the literature.

All retrieved records were imported into EndNote X9 (Clarivate Analytics) for deduplication. Two independent reviewers (CZ and HND) screened titles and abstracts against the eligibility criteria. Full-text articles of potentially relevant studies were retrieved and independently assessed. Discrepancies at each stage were resolved through discussion or, if necessary, adjudication by a third reviewer (LCX). The study selection process was documented using a PRISMA flow diagram (Figure 1).

A standardized, pilot-tested data charting form was developed in Microsoft Excel. Two reviewers independently extracted data from included studies. The form captured: (1) study characteristics (author, year, country, design); (2) participant characteristics (sample size, patient group); (3) intervention details (telemedicine modality/application platform, development method, core content elements, intervention frequency and duration, comparator); (4) outcome measures and assessment tools; and (5) key findings related to feasibility, effectiveness, and patient/caregiver experience. To move beyond a narrative listing and facilitate comparison across studies, extracted data were synthesized thematically. Findings were grouped by intervention modality, core content, and outcomes to identify patterns and gaps in the evidence base.

## 3 Results

### 3.1 Literature screening results

An initial search yielded 2,207 records. After multi-layered screening, 18 articles (12, 15–31) were ultimately included. The literature screening process is shown in Figure 1.

### 3.2 Basic characteristics of included studies

Among the 18 included articles, 2 were in Chinese (17, 19) and 16 in English (12, 15, 16, 18, 20–31).he included studies employed diverse methodological approaches. By primary study design, there were 5RCTs (15, 16, 24, 28, 31), 6 quasi-experimental studies (12, 17, 19, 20, 25, 26), 1 cohort study (29), and 6 studies that utilized a mixed-methods framework (18, 21–23, 27, 30). These mixed-methods studies integrated quantitative (e.g., surveys, outcome measures) and qualitative (e.g., interviews, focus groups) components to comprehensively evaluate the interventions. The basic characteristics of the included literature are presented in Table 1. In summary, the included studies, published between 2011 and 2025, originated from various countries (e.g., USA, Australia, Portugal, China, Austria) and employed a range of telemedicine modalities (apps, video platforms, integrated systems). Sample sizes were generally small to moderate. This diversity highlights the evolving and international nature of the field but also underscores the heterogeneity in intervention design and evaluation.

**Table 1:** Basic characteristics of the included studies (*n* = 18).

Author/publication time (year)	Sample size (test/control)	Development method	Application platform	Content elements	Intervention duration	Control group intervention method	Outcome measures
Mayan (15)/2025	45/45		Digital platform	Symptom assessment and tracking, advance care planning, communication aid, self-management strategies, resource linkage	14 weeks	Received usual care	A,B,G
Mayahara (16)/2025	131/131	Based on prior research, theoretical frameworks (e.g., symptom management theory), user involvement	e-PainSupport digital app	Pain assessment tools, symptom recording, personalized education, data feedback and reminders, provider-patient communication support	Follow-up until death	Received usual hospice pain management	B,D
Guo (17)/2024	34/33	Literature review, needs interviews, expert meetings, multidisciplinary team collaboration	WeChat mini-program	Patient side: personal data center, remote collaboration, instant empowerment. Provider side: Process management, intelligent alerts, data insights.	8 weeks	Received routine discharge guidance telephone follow-up	B,C,F
O’Connor (18)/2024	13		PalcareGo App	Symptom monitoring and management, patient empowerment, advance care planning, psychological support, provider coordination	21 months		A,B,C,G
Deng (19)/2023	80/80		Internet platform	Condition assessment, needs review, health consultation, psychological counseling, medication guidance	Received 3–5 sessions	Routine telephone follow-up	C,F
Jiang (20)/2023	7/21		WebRTC video communication platform	Symptom management, treatment advice, psychological support	3 months	Routine care	A,B,F,H
Gonzalez (21)_/_2023	15		APP	Symptom tracking, educational materials, communication tools, medication reminders	2–4 weeks		B,H
Stephens (22)/2022	6		VSee video conferencing platform	Structured shared decision-making, professional navigation & support, whole-family involvement	9 months, 20 min/session		H
Laranjeira (23)/2022	8	Needs assessment, qualitative interviews, focus groups, usability testing	mHealth App: Help2Care-PAL	Standardized symptom management, family psychosocial support, end-of-life planning and coordination, systematic health empowerment	4 weeks		B,E,F,H,I,J
Gatter (24)/2022	75/75		Telemedicine system	Remote consultation	24 months	Standard care first 12 months, switched to teleconsultation next 12 months.	A,B,C,D,F,H,I
Balasubramanian (25)/2022	120		Remote video e-PC software	Web-based application with registration, consultation, messaging, resource management functions			F
Begnoche (26)/2020	20		Commercial telemedicine platform	Symptom assessment and management, medication review and adjustment, care goal discussion and ACP, psychosocial and spiritual support, patient and family education	3 months		C,F,H
Nemecek (12)/2019	15	Technology integration, database construction	VSee App for secure video consultation	Video consultation, remote physiological monitoring, symptom & treatment diary, data visualization	Used until death		C,E,F,H
Bonsignore (27)/2018	101		TapCloud app	Symptom assessment, medication management, communication functions, family involvement, advance directives & palliative care referral	12 months		B,G,H
Hoek (28)/2017	36/38		Remote video consultation	Symptom assessment & management, problem & need identification, treatment policy discussion, multidisciplinary collaboration	Once weekly for 13 weeks	Received usual palliative care	B,E,F,I
Tieman (29)/2016	43		ntegrated telemedicine package (remote monitoring + care)	Video conferencing, patient self-report, caregiver self-report, remote activity monitoring, alert system, information resources	Mean 128.9 days (~4 months), range 17–415 days		F,H
Holland (30)/2014	29		Telemedicine device (with two-way video)	Symptom self-management, post-discharge remote follow-up, daily symptom screening	14 days		B,C,H
Duursma (31)/2011	50/50		Telemedicine application	Symptom assessment and management, psychosocial support, care coordination and continuity, family caregiver support, information provision and education	Weekly standardized teleconsultation	Routine home palliative care process	B,E,I

A, Healthcare resource utilization; B, Symptom burden; C, Quality of life; D, Functional status; E, Psychological state; F, Satisfaction; G, Care plan completion; H, Feasibility; I, Caregiver burden; J, Symptom management self-efficacy.

## 4 Discussion

### 4.1 Diversified development of telemedicine intervention models in HBPC

Telemedicine in home-based palliative care presents a pattern of diversified development. At the mobile application level, the Tap Cloud app integrated functions like symptom assessment, medication management, and real-time alerts (27). Dedicated device systems provided video consultation and physiological monitoring services *via* tablet devices pre-loaded with professional software (12). Intelligent integrated systems, leveraging platforms like We Chat Mini-Programs, incorporated AI analysis and automatic alert functions for personalized follow-up management (17). Specialty-specific platforms, such as those for neurological diseases, developed remote consultation systems with dual-network guarantees (24). Pain management applications established professional pain management systems through structured assessment and personalized education modules (16). These technological solutions demonstrate a complete developmental spectrum from general-purpose to specialized, and from basic functions to intelligent integration, offering diverse technical solutions for HBPC in different scenarios. In terms of service models, innovative developments have emerged, including the “clinic-to-home” hybrid care model (26), the “internet + offline service” integrated model (19), and disease-specific specialized service models (24). Regarding management strategies, a significant shift from standardization to personalization has been achieved. Demand-driven models (20) are gradually replacing fixed-frequency supply-driven models (28). Personalized management plans (16) and tiered alert systems (29) have enhanced service precision. Furthermore, the concept of integrated care has deepened. Multidisciplinary collaboration models (27), caregiver support systems (23), and comprehensive management systems (18) jointly construct a holistic remote palliative care service network. This diversified landscape reflects both the inclusivity and adaptability of technological applications and highlights a patient-needs-centered service philosophy, providing varied and practical solutions for home-based palliative care in different contexts.

### 4.2 Feasibility, effectiveness, and cost-effectiveness of telemedicine in HBPC applications

The application of telemedicine demonstrates clear advantages, capable of overcoming time and space constraints, contributing to improved care quality and efficiency, and reducing healthcare costs (32). Regarding feasibility, multiple studies confirmed its potential for technical implementation and service delivery. Practice showed that integrating remote services into routine outpatient workflows could successfully establish a “clinic-to-home” continuous care model, proving its operability within existing healthcare systems (26). In terms of technology acceptance, mobile application-based remote systems were confirmed to have high usability and user acceptance. Users generally found them easy to operate, responsive, and effective in enhancing the efficiency and quality of care services (27). Even in environments with weak technological infrastructure, such as nursing homes, telemedicine could be accepted and used by different groups with appropriate technical support (22). Regarding effectiveness, telemedicine demonstrated multi-faceted clinical value, Future meta-analyses on this topic would benefit from careful consideration of appropriate effect size measures (e.g., standardized mean differences for continuous outcomes) and model selection (fixed vs. random effects), alongside reporting heterogeneity metrics (e.g., I^2^ statistic) to better quantify and interpret the pooled evidence. Including symptom management, quality of life improvement, and psychological state enhancement. Research indicated that mobile app-based remote interventions could effectively alleviate patient symptoms such as pain, dyspnea, and depression, and significantly improve overall quality of life (27). Another study noted that although patients using a specific app might experience short-term fluctuations in emotional well-being, their overall quality of life still showed a positive improvement trend (18). Furthermore, remote support also demonstrated positive effects in providing psychological support and guidance, effectively reducing patients’ anxiety and depression levels and offering continuous emotional support (12). In terms of cost-effectiveness, telemedicine demonstrated significant benefits in reducing medical costs and optimizing resource allocation. Studies showed this model could substantially lower the direct financial burden on patient families (25). Through early symptom intervention and continuous monitoring, telemedicine significantly reduced unplanned emergency visits and hospitalizations due to worsening conditions (18, 20, 24, 26). This proactive health management approach not only avoided unnecessary medical expenditures but also improved the efficiency of healthcare resource utilization, allowing limited resources to serve the patients in greatest need more precisely, thereby achieving dual benefits at both individual and societal levels.

### 4.3 Challenges and recommendations for telemedicine application in HBPC patients

Currently, telemedicine, as a feasible and promising technology, has seen preliminary application in home-based palliative care patients. However, several constraining factors require attention and improvement.

1. *Challenges related to the target population*: Patients receiving HBPC are typically older adults at the end of life, often experiencing physical frailty and cognitive decline. These factors may impact their ability to learn and their willingness to adopt remote technologies. Therefore, it is recommended to conduct a comprehensive assessment of older patients’ acceptance levels and physical status prior to implementing telemedicine services. In designing remote systems, patient-facing interfaces must be simple, clear, and intuitive. Equal emphasis should be placed on enhancing both age-friendly design and humanistic care in content to improve the patient experience (21). When promoting remote technologies, the role of multidisciplinary teams should be fully leveraged to strengthen technical training and ongoing support for patients, caregivers, and their families.
2. *Lack of standardization in research and practice*: Existing studies lack unified norms regarding intervention frequency, duration, and evaluation criteria. It is recommended to establish a standardized framework for remote palliative care services based on evidence-based medicine, while maintaining flexibility to adapt to the personalized needs of different patients. Furthermore, there is a need to improve service quality evaluation systems, incorporating multi-dimensional indicators such as patient experience, family burden, and cost-effectiveness to provide a basis for service optimization.
3. *Shortage of professional palliative care teams*: The integration of telemedicine into HBPC services faces challenges stemming from a structural deficit in professional expertise. Key issues include a shortage of healthcare personnel who have undergone systematic palliative care training (33), and a scarcity of versatile talents (professionals with integrated competencies in both specialized care and telemedicine technology application). At present, palliative care education and research in China predominantly concentrate on training medical staff, whereas public education efforts have not yet formed a complete framework (34). Consequently, China needs to proactively learn from international advanced models in palliative care education to expedite the cultivation of professionals possessing solid theoretical foundations and extensive clinical practical skills (35). In parallel, service standards, technical guidelines, and workflows for HBPC must be further enhanced. Systematic training should be consistently implemented to strengthen the home-based healthcare delivery capabilities of providers, thereby ensuring patients receive high-quality home-based palliative care (36).
4. *Need for exploration of applicability and cultural adaptation*: Future research should more thoroughly investigate the applicability and cultural adaptation of intervention models. This review identified that different remote intervention models are suited to distinct scenarios: applications and web-based platforms are particularly effective for continuous monitoring and structured management, such as symptom tracking and medication reminders; video conferencing is indispensable for facilitating complex shared decision-making and providing emotional support; models based on social platforms like WeChat hold significant potential for enhancing accessibility and widespread adoption. Further research is needed to clarify which patient populations, disease stages, and clinical objectives each model is best suited for. Crucially, service models must also align with Chinese family cultural norms. System designs should incorporate dedicated access for caregivers to facilitate condition reporting and support seeking. Support content should combine skill training with stress-relief resources to mitigate the burden associated with filial responsibilities. Communication processes should encourage whole-family participation in decision-making through video conferences, thereby seamlessly integrating traditional family caregiving models into remote care scenarios and fostering more humane, culturally resonant care models.
5. *Methodological limitations of current research*: Most studies on the application of telemedicine in HBPC suffer from small sample sizes and relatively homogeneous research designs. It is recommended that future studies conduct more high-quality, large-sample, multicenter randomized controlled trials to further validate the effects of remote interventions in different types of palliative care patients, including their applicability and effectiveness within specific healthcare environments such as China’s.

### 4.4 Implications for future development and specific considerations for the Chinese context

Building on the synthesized evidence and identified challenges, the future development of telemedicine in HBPC should prioritize several areas, with particular adaptations needed for systems like China’s.

First, there is a pressing need for standardized yet adaptable service frameworks. This involves developing national guidelines that define core service components, technical standards, and quality metrics for tele-HBPC, while allowing for regional customization to address local resource disparities and patient needs. Second, addressing the workforce shortage is critical. This requires integrating competencies in both palliative care and telemedicine into the core curricula of medical and nursing education, alongside creating up skilling programs for existing healthcare professionals. Third, technology design and service delivery models must achieve cultural congruence. In family-centric societies like China, digital platforms should be designed to facilitate and empower family involvement in care coordination, communication, and decision-making, rather than aiming to replace the familial caregiving role. Fourth, equitable access must be ensured. Strategies should focus on improving digital infrastructure in underserved areas and providing tailored support to enhance digital literacy among older patients and their families, to prevent the widening of health disparities. Finally, sustainable financing models must be explored. Incorporating tele-HBPC into basic medical insurance schemes, allocating public health funds for pilot programs, and fostering public-private partnerships are potential pathways to ensure the long-term viability and accessibility of these services.

## 5 Summary

This scoping review synthesizes the evidence on telemedicine applications in home-based palliative care (HBPC), examining their development, core components, outcomes, and overall effects. The findings indicate that, through diverse technological platforms and personalized service models, telemedicine yields significant benefits in symptom management, quality-of-life enhancement, and healthcare resource optimization, demonstrating clear feasibility and cost-effectiveness. However, significant gaps and persistent challenges remain, particularly in cultural adaptability (especially within family-centric contexts such as China’s), localized evidence, service standardization, age-adapted design, and professional capacity building. To advance the field toward standardized and scalable implementation, especially in contexts like China, future initiatives must prioritize high-quality, context-specific research, strengthen interdisciplinary workforce training, enhance supportive policy frameworks, and engage in co-designed, culturally congruent implementation strategies. These concerted efforts are essential to support the establishment of a sustainable, high-quality home-based palliative care system that can effectively meet localized needs and contexts.

## Data Availability

The original contributions presented in the study are included in the article/supplementary material, further inquiries can be directed to the corresponding author.
