# Correction: A scoping review of the application of telemedicine in home-based palliative care patients: insights for healthcare systems with emphasis on the Chinese context

**DOI:** 10.3389/fpubh.2026.1830130

**Published:** 2026-03-17

**Authors:** Frontiers Production Office

**Affiliations:** Frontiers Media SA, Lausanne, Switzerland

**Keywords:** home-based care, hospice care, palliative care, scoping review, telecare, telemedicine

Author Dongying Li was erroneously assigned to affiliation 2 “School of Nursing, Jiangxi Medical College, Nanchang University, Nanchang, China”. This affiliation has now been removed for author Dongying Li. The author should be affiliated with affiliation 1 “Department of Intensive Care Unit, The Second Affiliated Hospital of Nanchang University, Nanchang, China” instead.

The original version of this article has been updated.

